# Melanoma-associated mutants within the serine-rich domain of PAK5 direct kinase activity to mitogenic pathways

**DOI:** 10.18632/oncotarget.25356

**Published:** 2018-05-22

**Authors:** Kyle M. LaPak, Dennis C. Vroom, Ayush A. Garg, Xiangnan Guan, John L. Hays, Jonathan W. Song, Christin E. Burd

**Affiliations:** ^1^ Department of Molecular Genetics, The Ohio State University, Columbus, OH, USA; ^2^ Department of Mechanical and Aerospace Engineering, The Ohio State University, Columbus, OH, USA; ^3^ Division of Medical Oncology, Department of Internal Medicine, The Ohio State University, Columbus, OH, USA; ^4^ Department of Cancer Biology and Genetics, The Ohio State University, Columbus, OH, USA

**Keywords:** PAK7, PKA, melanoma, melanocyte, p21-activated kinase

## Abstract

The overexpression and hyperactivity of p21-activated serine/threonine kinases (PAKs) is known to facilitate tumorigenesis; however, the contribution of cancer-associated *PAK* mutations to tumor initiation and progression remains unclear. Here, we identify p21-activated serine/threonine kinase 5 (PAK5) as the most frequently altered PAK family member in human melanoma. More than 60% of melanoma-associated *PAK5* gene alterations are missense mutations, and distribution of these variants throughout the protein coding sequence make it difficult to distinguish oncogenic drivers from passengers. To address this issue, we stably introduced the five most common melanoma-associated *PAK5* missense mutations into human immortalized primary melanocytes (hMELTs). While expression of these mutants did not promote single-cell migration or induce temozolomide resistance, a subset of variants drove aberrant melanocyte proliferation. These mitogenic mutants, PAK5 S364L and D421N, clustered within an unstructured, serine-rich domain of the protein and inappropriately activated ERK and PKA through kinase-independent and -dependent mechanisms, respectively. Together, our findings establish the ability of mutant PAK5 to enhance PKA and MAPK signaling in melanocytes and localize the engagement of mitogenic pathways to a serine-rich region of PAK5.

## INTRODUCTION

Alterations in *BRAF*, *NRAS*, and *NF1* define the canonical genetic subtypes of melanoma, yet each of these oncogenic mutations is insufficient to drive tumorigenesis [1]. Only a fraction of BRAF- or NRAS-mutant melanocytes form tumors in genetically engineered mouse models, and benign human nevi harboring these oncogenic lesions rarely progress to form tumors [2-10]. Drugs targeting alterations in *BRAF*, *NRAS*, or *NF1* are either unavailable or fail to elicit durable responses [11-16]. Thus, there is an ongoing need to elucidate new, therapeutically tractable genetic alterations prevalent in melanoma. Unfortunately, efforts to identify such targets have been hindered by the inordinate number of somatic gene variants in human melanomas [1, 17-19]. Here, we define the oncogenic potential of common missense mutations in the p21-activated kinase (PAK) family of serine/threonine kinases, revealing new insights relevant to the therapeutic targeting of these proteins in melanoma and other tumor types.

PAKs are classified into two groups based upon peptide sequence homology [20]. Group I PAKs (PAK1, 2, and 3) contain an N-terminal p21 binding domain (PBD) which mediates interactions with Rho family GTPases such as RHO, RAC, and CDC42 [21-23]. Binding of Rho family members to the PAK PBD promotes kinase activity by alleviating inhibitory, intermolecular interactions between the PAK N-terminal autoinhibitory (AID) and C-terminal kinase domains [21-23]. Mechanism governing the kinase activity of group II PAKs (PAK4, 5/7, 6) are still under investigation, with two distinct regulatory models proposed. In the first model, derived from studies of PAK4 purified from mammalian cells, N-terminal CDC42 binding is sufficient to displace the group II AID and stimulate kinase activation [24]. However, in the second model, based upon experiments employing mammalian PAK4 isolated from bacteria, it is suggested that maximal catalytic activity is achieved only when CDC42 engagement is accompanied by binding of SH3 domain-containing proteins to an N-terminal pseudosubstrate domain present in group II PAKs [25]. Once activated, all PAKs phosphorylate a myriad of downstream targets, but can also exhibit kinase-independent scaffolding functions that enhance cellular signal transduction. For instance, PAK1 facilitates the interaction of proteins within the MAPK and PI3K/AKT cascades, leading to increased activation of these pathways [26-32]. While reports of such kinase-independent functions are common for group I PAKs, similar activities have yet to be fully explored in group II PAKs.

PAKs control many cellular processes implicated in cancer initiation and progression, including: cytoskeletal remodeling, cell survival, and proliferation [33]. Alterations that increase PAK levels, such as genomic amplification or mRNA/protein overexpression, occur frequently in cancer and contribute to tumor progression [33]. For example, *PAK1* amplification drives the anchorage-independent growth of breast cancer cell lines, while duplications of *PAK1* and 4 correlate with poor outcomes in patients with ovarian cancer [34-36]. Elevated PAK1 protein levels are associated with colorectal, hepatocellular, and prostate cancer metastasis, and PAK2 and 6 overexpression is linked to chemotherapeutic and radiation resistance in breast and prostate cancers, respectively [37-42]. These findings have stimulated interest in developing ATP-competitive compounds to limit PAK kinase activity; yet, the development of specific inhibitors has been impeded by the large and flexible PAK catalytic pocket [43]. The only PAK inhibitor to reach a phase I clinical trials failed due to low oral bioavailability, high toxicity, and minimal tumor responses [43, 44].

Few studies have investigated the role of PAKs in melanoma. These limited reports in melanoma cell lines and mouse models, associate enhanced PAK1 and 4 activity with aberrant proliferation, invasion, and therapeutic resistance [45-48]. Heightened PAK1 signaling is also associated with aquired resistance to single-agent BRAF inhibitor and dual-agent BRAF/MEK inhibitor therapies [49, 50]. Such work establishes a role for heightened *PAK* activity in melanoma progression and therapeutic resistance, yet studies investigating the oncogenic potential of *PAK* missense mutations are lacking. Here, we elucidate mechanisms by which common *PAK* missense mutations drive inappropriate melanocyte proliferation, revealing functional insights relevant to the formulation of drugs to target PAKs in cancer.

## RESULTS

### *PAK5* is the most frequently altered *PAK* family member in human melanoma

**Figure 1:**
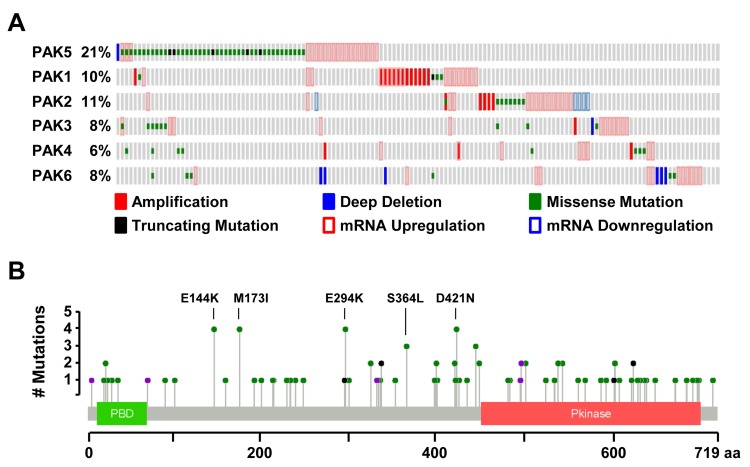
PAK5 is the most frequently altered PAK family member in melanoma **A.** Oncoprint generated in cBioPortal depicting alterations in the *PAK* gene family for 287 melanomas collected by TCGA (provisional data accessed on 08/2016). PAKs are divided into two groups based upon protein sequence homology: group I (PAK1, PAK2 and PAK3) and group II (PAK4, PAK5, and PAK6). **B.** Protein diagram of human PAK5 depicting the location and frequency of melanoma-associated variants. Green dots represent missense mutations, black dots represent truncating mutations, and purple dots represent splice-site mutations.

Using cBioportal, we examined the alteration status of all six *PAK* genes in a dataset of 287 melanoma samples curated by The Cancer Genome Atlas (TCGA) Research Network [51, 52]. Within these samples, *PAK5* alterations were nearly twice as frequent as those observed in other *PAK* family members (21% versus 6-11%; Figure 1A). This enrichment was unique to melanoma, as *PAK5* alterations were infrequent in other TCGA-analyzed tumor types (<9%; Supplementary Figure 1A). Most melanoma-associated *PAK5* alterations were missense mutations (62%), localized throughout the protein coding sequence within both the PBD and kinase domain as well as uncharacterized regions (Figure 1A, 1B). We investigated the co-occurrence of *PAK5* alterations with the three most common melanoma drivers: *BRAF*, *NRAS*, and *NF1*. While *PAK5* alterations were randomly distributed across both *BRAF*- and *NRAS*-mutant melanomas (*p* = 0.20 and 0.30, respectively), these alterations were significantly enriched in *NF1*-mutant tumors (*p* = 0.01; Supplementary Figure 1B, 1C). *PAK5* alterations did not co-occur with other group II *PAKS*, but instead, coincided with alterations in *PAK3* (*p* = 0.02; Figures 1A, Supplementary Figure 1D). Taken together, these data show that *PAK5* is the most frequently mutated *PAK* in human melanoma.

### Melanoma-common *PAK5* mutations do not alter PAK5 kinase activity

**Figure 2:**
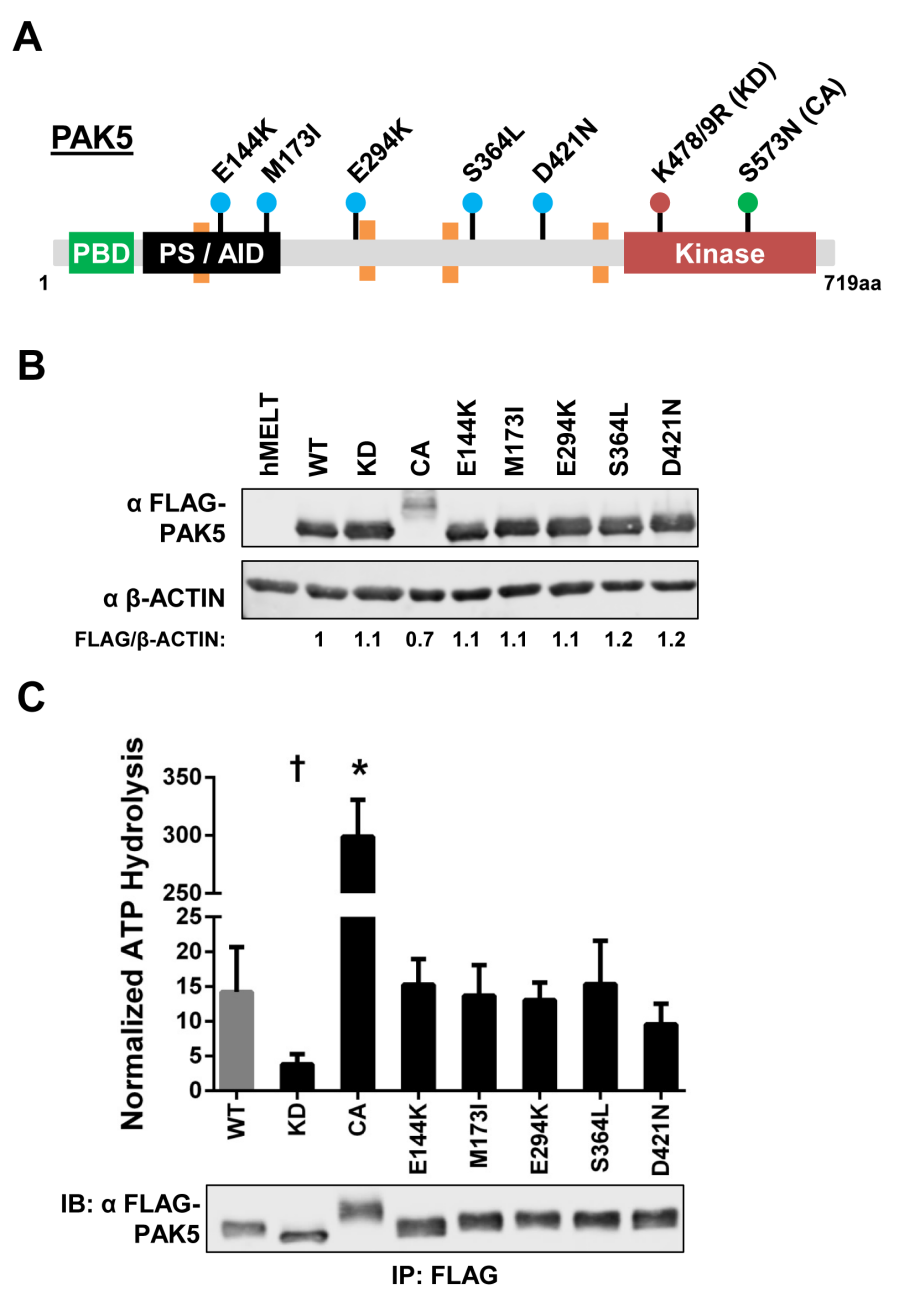
Melanoma-common PAK5 mutants do not alter kinase activity **A.** Protein diagram depicting the location of human PAK5 variants examined in this study. Dots are color-coordinated to divide the mutations into groups: the most frequent melanoma variants (E144K, M173I, E294K, S364L and D421N) in blue, a kinase dead mutant (K478/9R, KD) in red, and a constitutive active mutant (S573N, CA) in green. PBD = p21-binding domain, PS/AID = pseudosubstrate/auto-inhibitory domain, Kinase = kinase domain. Orange boxes represent proline-rich motifs (PxxP). **B.** Immunoblot showing the stable expression of FLAG-tagged PAK5 variants in immortalized human primary melanocytes (hMELTs). ‘hMELT’ indicates the parental cell line. Shown below each lane is the β-ACTIN-normalized level of mutant PAK5 expression relative to PAK5 wildtype (WT). **C.** PAK5 kinase activity was measured using the ADP-Glo system and normalized to immunoprecipitated protein levels as measured by immunoblot. A representative immunoblot of immunoprecipitated FLAG-PAK5 is shown. Each bar represents three biological replicates with the mean and standard deviation indicated. † = *p* < 0.05, * = *p* < 0.001 compared to PAK5 WT (gray bar), where *p*-values were calculated using multiple t-tests and significance determined by FDR (Q = 5%).

PAK5 missense mutations are evenly distributed throughout characterized and uncharacterized domains of the protein, making it difficult to predict the functional consequences of these alterations in melanoma (Figure 1B). Therefore, we selected the five most frequently observed PAK5 mutants in our dataset for further analysis: E144K, M173I, E294K, S364L, and D421N (Figures 1B, 2A; blue dots). A kinase-dead *PAK5* mutant (K478/9R, KD) and a constitutive active *PAK5* mutant (S573N, CA) were included as controls in our experiments (Figure 2A; red and green dots, respectively) [24, 53]. FLAG-tagged versions of these mutant proteins were stably expressed in human primary immortalized melanocytes (hMELTs) under the control of a *UbC* promoter. FLAG-PAK5 expression was consistent amongst the stable cell lines except for *PAK5* CA, which displayed ∼30% lower protein levels (Figure 2B). We also observed that PAK5 CA migrated at a higher molecular weight, presumably due to protein post-translational modifications (Figure 2B).

Pre-clinical therapeutics target PAK kinase activity [43]. To determine if common melanoma-associated PAK5 mutations affect enzymatic function, we immunoprecipitated individual *PAK5* mutants from our hMELT stable cell lines and subjected these proteins to *in vitro* kinase assays using a canonical PAK4 peptide substrate [54]. As expected, *PAK5* KD displayed dramatically reduced kinase activity, while PAK5 CA increased peptide phosphorylation >20-fold over wildtype PAK5 (Figure 2C). Kinase activity of the melanoma-associated PAK5 mutants, E144K, M173I, E294K, S364L, and D421N, was similar to the wildtype control (Figure 2C). These data demonstrate that the protein products of melanoma-common *PAK5* missense mutations do not exhibit altered kinase activity.

### Single-cell melanocyte migration and temozolomide resistance are unchanged by the expression of melanoma-common PAK5 mutants

**Figure 3:**
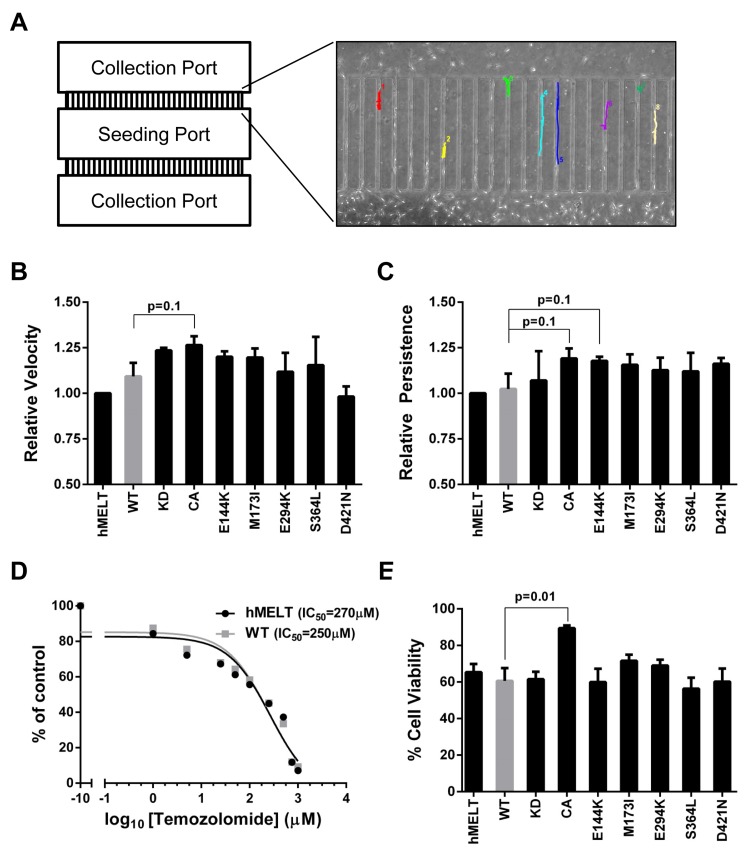
Melanoma-associated PAK5 mutants do not affect *in vitro* melanocyte migration or resistance to temozolomide **A.** hMELT stable cell lines were placed in the seeding port of the depicted microfluidics device. These cells were serum-starved while full serum medium was added to the flanking collection ports to stimulate migration. Using time-lapse microscopy, images were taken every five minutes for a total of twelve hours. Shown is a representative image of cell tracking through the microfluidics device channels using ImageJ. **B.** The average speed (µm/min) at which hMELT stable cell lines traversed the microfluidics device is shown relative to the parental line. Each bar represents >130 analyzed cells across at least three biological replicates with standard error of the mean indicated. *p*-values were calculated using multiple t-tests and significance determined by FDR (Q = 5%). **C.** The ability of single cells to persistently migrate in one direction is shown for each PAK5 mutant relative to the parental line. Each bar represents >130 analyzed cells across at least three biological replicates with standard error of the mean indicated. *p*-values were calculated using multiple t-tests and significance determined by FDR (Q = 5%). **D.** hMELT parental and wildtype (WT) PAK5 cells were seeded in a 96-well plate and treated with various concentrations of temozolomide ranging from 0 µM to 1 mM. Treatment media was changed every two days for a total of seven days after which cell viability was measured using resazurin metabolism. The IC_50_ was determined using the ‘log(inhibitor) vs. response (three parameters)’ analysis in GraphPad Prism software. **E.** hMELT-PAK5 stable cell lines were seeded in a 96-well plate and treated with either 250 µM temozolomide or DMSO. The treatment media was changed every two days for a total of seven days. Cell viability was measured using resazurin metabolism and normalized to the DMSO control. ‘hMELT’ indicates the parental cell line. Each bar represents five biological replicates performed in triplicate with the mean and standard error of the mean indicated. *p*-values were calculated using multiple t-tests and significance determined by FDR (Q = 5%).

PAK proteins regulate a variety of cytoskeletal remodeling processes critical for tumor migration and invasion [55]. To examine the impact of *PAK5* mutations on melanocyte migration, we used time-lapse microscopy and measured the motility of our hMELT stable cell lines across a microfluidics device (Figure 3A, Supplemental Movie). This microfluidics assay provides several advantages over traditional measures, such as Boyden chamber or scratch assays. The device allows for the simultaneous tracking of bi-directional cell velocity and migratory persistence. Furthermore, confounding survival or proliferation differences are eliminated because data is only acquired from viable, non-dividing cells. To conduct this assay, we seeded our hMELT stable cell lines under full serum conditions into the central port of the device. The following day, cells within the seeding port were starved of growth factors, while full serum media was kept in the flanking collection ports. After 36 hours, images were taken at five minute intervals over a 12 hour period and stitched together using ImageJ. We measured the velocity and directional persistence of single cells migrating through the device channels. hMELT cell lines harboring wildtype or mutant PAK5 exhibited a similar velocity; however, expression of PAK5 CA stimulated a marginal increase in migratory speed (*p* = 0.1; Figure 3B). The directional persistence of PAK5-mutant hMELTs was also unchanged in comparison to cells harboring wildtype PAK5, but trended towards significance in both the PAK5 CA and PAK5 E144K-expressing cell lines (*p* = 0.1; Figure 3C). These data show that common, melanoma-associated PAK5 mutants do not drastically change the single-cell migratory velocity or persistence of immortalized melanocytes.

Exogenous expression of wildtype PAK5 was shown to increase apoptotic resistance in CHO cells treated with camptothecin or C2-ceramide [56]. To determine whether melanoma-common PAK5 mutants confer resistance to temozolomide, an alkylating chemotherapeutic drug approved for the treatment of malignant melanoma [57], we first ascertained the IC_50_ of temozolomide in hMELT cells expressing wildtype PAK5 (250 µM; Figure 3D). The viability of our hMELT stable lines was assessed after seven days of treatment with 250 µM temozolomide using both resazurin metabolism and direct cell counts (Figure 3E, data not shown). While the expression of melanoma-common PAK5 mutants had no effect on temozolomide-treated cells, evidence of resistance was observed in melanocytes harboring PAK5 CA (*p* = 0.01; Figure 3E).

### PAK5 S364L and D421N enhance melanocyte proliferation

**Figure 4:**
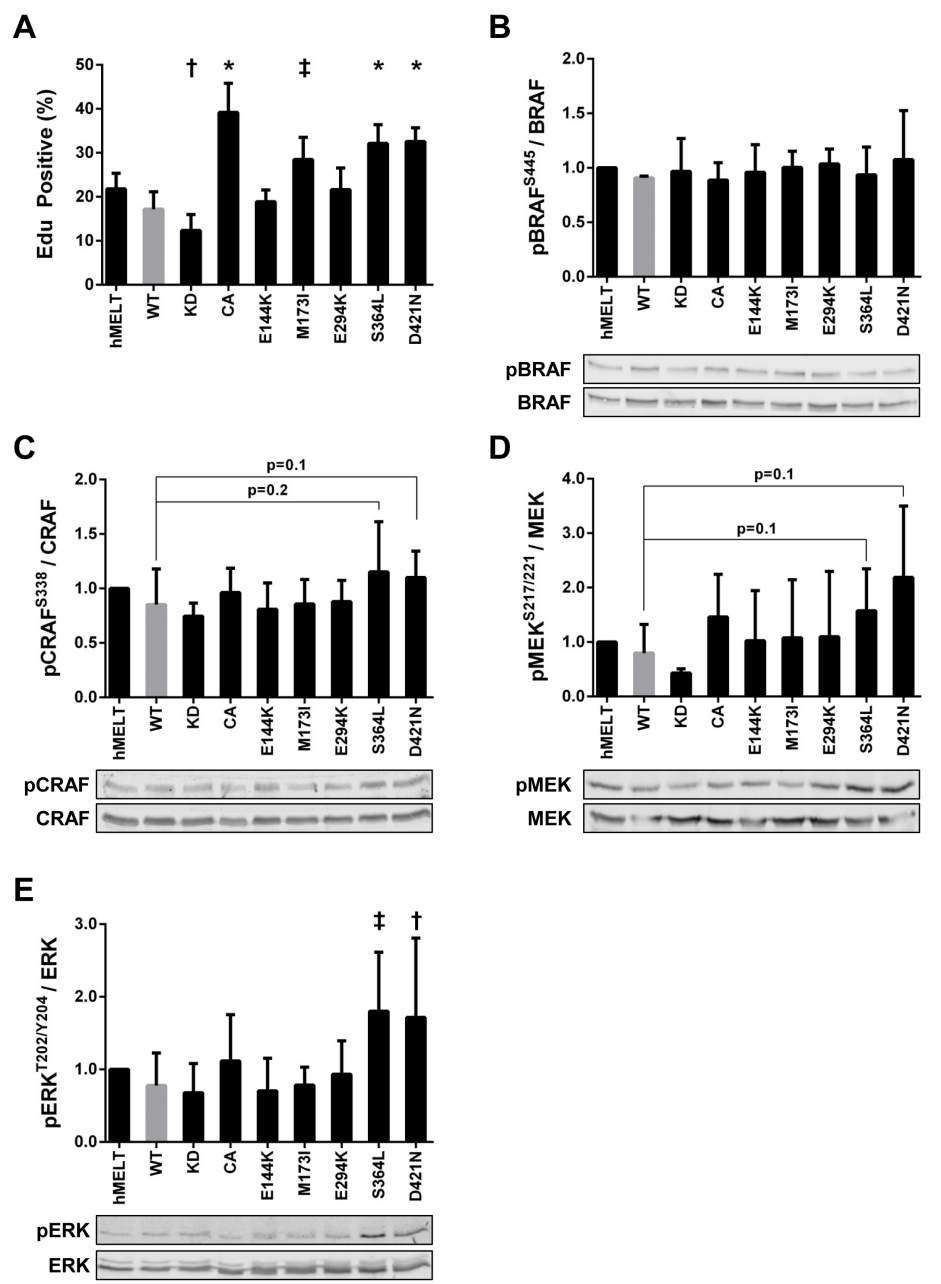
PAK5 S364L and D421N promote melanocyte proliferation and ERK activation **A.** hMELT stable cell lines were placed in 2% serum for 18 hours and then labeled with EdU for eight hours. After labeling, cells were fixed for flow cytometric analysis. Shown is the average percentage of EdU positive cells across at least three biological replicates with error bars representing the standard deviation. † = *p* < 0.05, ‡ = *p* < 0.01, * = *p* < 0.001 compared to PAK5 WT (gray bar), where *p*-values were calculated using multiple t-tests and significance determined by FDR (Q = 5%). ‘hMELT’ indicates the parental cell line. **B.**-**E.** Lysates from hMELT stable cell lines, incubated in 2% serum as described in ‘A’, were analyzed by SDS-PAGE followed by immunoblotting. Bar graphs show phospho-protein levels normalized to total protein and relative to parental hMELT cells. At least four biological replicates were run with the mean and standard deviation indicated. A representative immunoblot is shown below each graph. † = *p* < 0.05, ‡ = *p* <0.01, compared to PAK5 WT (gray bar), where *p*-values were calculated using multiple t-tests and significance determined by FDR (Q = 5%).

**Figure 5:**
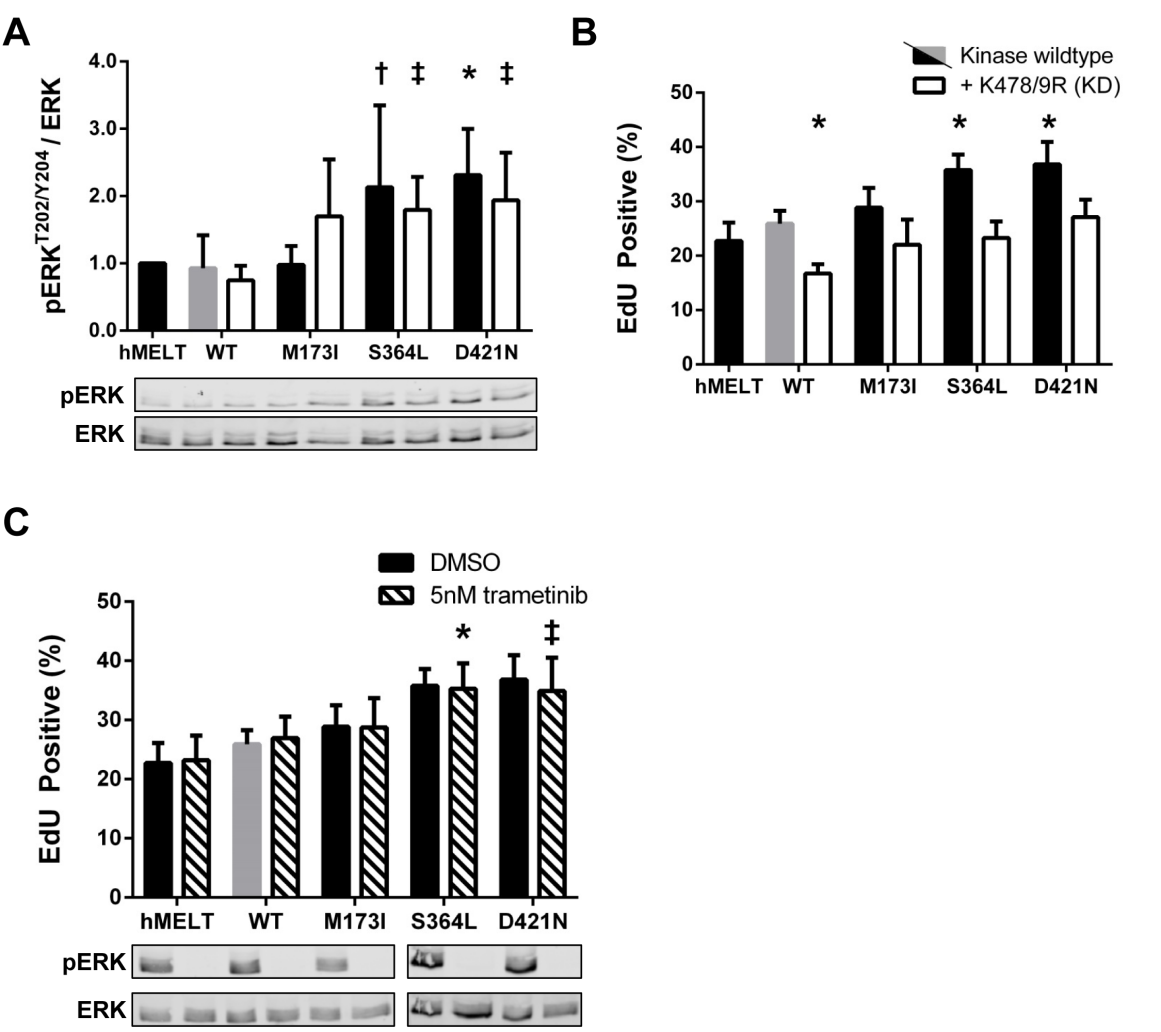
Kinase-independent functions of PAK5 S364L and D421N activate ERK but do not promote proliferation **A.** Lysates from hMELT stable cell lines, incubated in 2% serum for 18 hours, were analyzed by SDS-PAGE followed by immunoblotting. Bar graphs show phospho-protein levels normalized to total protein and relative to parental hMELT cells. At least four biological replicates were run with the mean and standard deviation indicated. A representative immunoblot is shown below. † = *p* < 0.05, ‡ = *p* <0.01, * = *p*<0.001 compared to PAK5 WT (gray bar), where *p*-values were calculated using multiple t-tests and significance determined by FDR (Q = 5%). **B.** The proliferation of hMELT stable cell lines was analyzed as in Figure 4A. Shown is the average percentage of cells staining EdU positive across at least three biological replicates with error bars representing the standard deviation. * = *p* < 0.001 compared to PAK5 WT (gray bar), where *p*-values were calculated using multiple t-tests and significance determined by FDR (Q = 5%). **C.** hMELT stable cell lines were treated with 5nm trametinib 30 minutes prior to the addition of EdU. Shown is the average percentage of cells staining EdU positive across at least three biological replicates with error bars representing standard deviation. † = *p* < 0.05, ‡ = *p* < 0.01, compared to trametinib-treated PAK5 WT (dashed gray bar), where *p*-values were calculated using multiple t-tests and significance determined by FDR (Q = 5%). ‘hMELT’ indicates the parental cell line.

**Figure 6:**
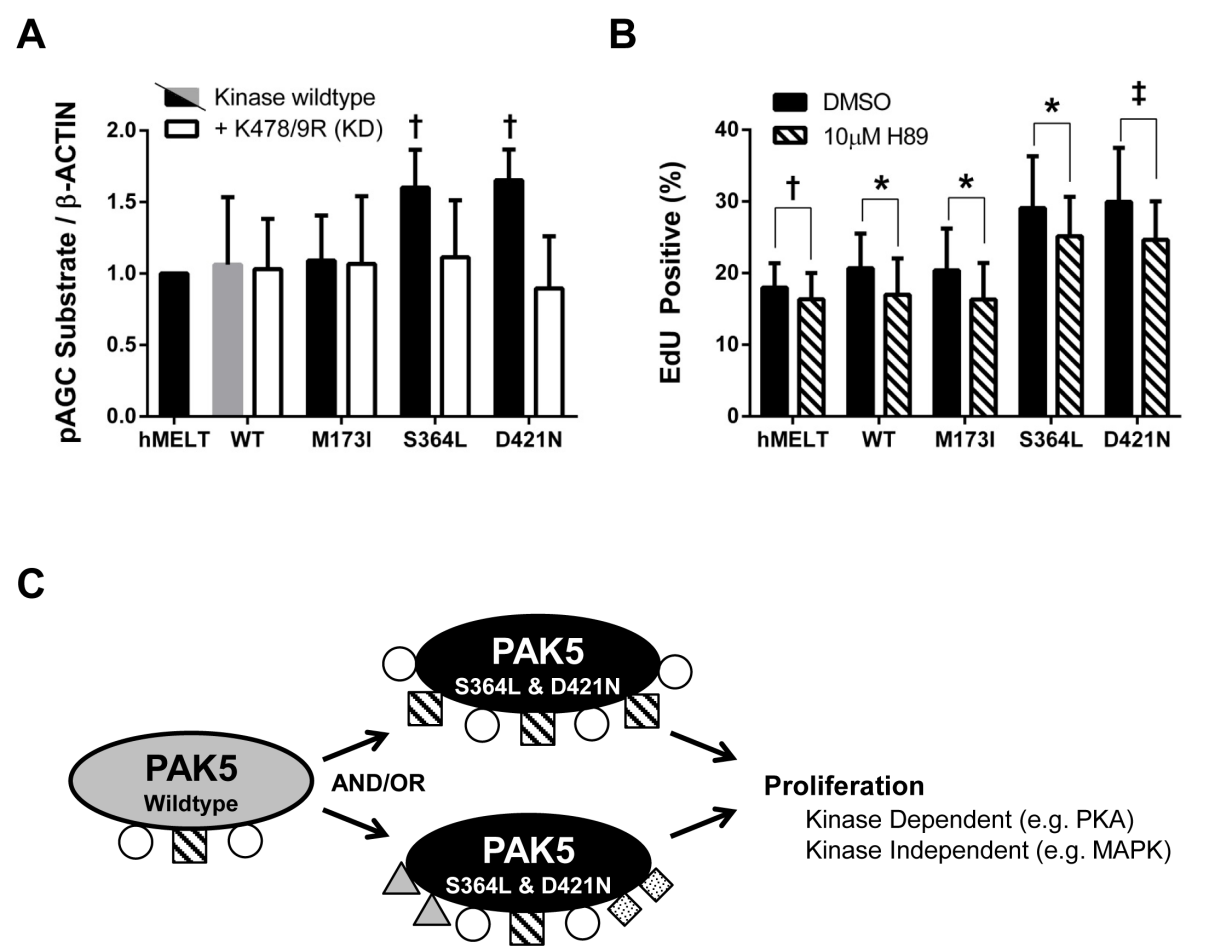
PAK5 S364L and D421N promote proliferation through PKA activation **A.** Lysates from hMELT stable cell lines, incubated in 2% serum for 18 hours, were analyzed by SDS-PAGE followed by immunoblotting with an antibody that recognizes the phosphorylated substrate motif of AGC kinases. ‘hMELT’ indicates the parental cell line. Each bar indicates the average, β-ACTIN-normalized, pAGC substrate signal relative to parental hMELT cells across at least three biological replicates. Error bars represent the standard deviation. † = *p* < 0.05 compared to PAK5 WT (gray bar), where *p*-values were calculated using multiple t-tests and significance determined by FDR (Q = 5%). **B.** hMELT stable cell lines were treated with 10 µM H89 or vehicle (DMSO) 30 minutes prior to the addition of EdU. Shown is the average percentage of EdU positive cells across at least three biological replicates with error bars representing the standard deviation. ‘hMELT’ indicates the parental cell line. † = *p* < 0.05, ‡ = *p* < 0.01, * = *p* < 0.001 compared to PAK5 WT (gray bar), where *p*-values were calculated using a paired t-test. **C.** Model showing the proposed mechanism by which PAK5 S364L and D421N promote melanocyte proliferation.

In addition to cellular migration and apoptotic resistance, PAKs are frequently implicated in proliferative control [58]. Therefore, we examined the effect of each PAK5 mutant on melanocyte proliferation. We seeded our hMELT stable cell lines at 40-50% confluence. The next day, the cells were placed in media containing 2% serum. After 18 hours, these cultures were pulsed with EdU for eight hours to monitor cellular proliferation Using flow cytometry to quantify EdU incorporation, we found that cells harboring PAK5 CA displayed increased proliferation, while those containing the PAK5 KD showed decreased EdU incorporation, as compared to wildtype PAK5 (Figure 4A). Notably, two of the melanoma-associated mutants, S364L and D421N, promoted melanocyte proliferation (87% and 89% increase, respectively; Figure 4A). A modest increase in proliferation was also observed with PAK5 M173I; however, this increase did not reach statistical significance in subsequent assays (See Figures 4A, 5B, 6B).

PAK1, 3, and 5 are reported to interact directly with CRAF and/or MEK to stimulate growth factor-independent proliferation [1, 59-64]. To examine MAPK signaling in our hMELT stable cell lines, we first assessed the phosphorylation status of the RAF homologs, BRAF and CRAF. Levels of BRAF-pS445 remained unchanged in all the hMELT stable cell lines (Figures 4B). In contrast, CRAF-pS338 increased moderately in PAK5 S364L and D421N-expressing melanocytes (*p* = 0.1, *p* = 0.2, respectively; Figure 4C). Because there was little change in RAF activation, we investigated downstream MAPK targets. Increases in MEK activation were highly variable in hMELT cells stably expressing PAK5 S364L or D421N and did not reach statistical significance (*p* = 0.1; Figure 4D). However, a more striking phenotype was observed downstream of MEK, where expression of either PAK5 S364L and D421N nearly doubled ERK-pT202/204 levels in comparison to the wildtype control (*p* = 0.005 and *p* = 0.04, respectively; Figure 4E). Together, these data correlate ERK activation with the enhanced proliferation seen in hMELT cells expressing PAK5 S364L or D421N.

### PAK5 S364L and D421N promote melanocyte proliferation dependent on kinase activity

PAK1 is reported to activate MAPK signaling through both kinase-dependent and -independent mechanisms [32, 59-61]. Therefore, we generated hMELT stable cell lines harboring kinase-dead (K478/9R; KD) versions of the PAK5 M173I, S364L, and D421N mutants, and confirmed the expression and catalytic deficiency of these proteins (Supplementary Figure 2A, 2B). Stable expression of PAK5 S364L-KD or D421N-KD increased pERK-T202/Y204 levels nearly two-fold over wildtype PAK5 (Figure 5A), recapitulating the phenotype of the kinase-proficient mutants. However, expression of PAK5 S364L-KD or D421N-KD did not enhance melanocyte proliferation as measured by EdU incorporation, suggesting that hMELT cells are insensitive to ERK-mediated mitogenic signals (Figure 5B). Indeed, treatment of parental and PAK5-expressing hMELT cells with a selective MEK inhibitor, trametinib, completely abolished ERK activity in these cells without altering cellular proliferation (Figure 5C). These data demonstrate that although PAK5 S364L-KD and D421N-KD enhance ERK activity in a kinase dependent manner, this mechanism is not responsible for the increased mitogenic activity of select PAK5 mutants in hMELT cells.

### Activation of PKA by PAK5 S364L and D421N promotes proliferation

The finding that hMELT cells grow in a MAPK-independent manner was not surprising. hMELTs are immortalized with both hTERT and CDK4^R24C^, and prior studies establish the ability of constitutive CDK4/Cyclin D1 activity to drive MAPK-independent melanoma growth [65-67]. However, because the p16-CDK4/6-Rb axis is deregulated in ∼70% of melanomas [1], our hMELT stable lines provided an excellent backdrop for the discovery of clinically relevant pathways engaged by mitogenic PAK5 mutants. We began by examining the WNT and cAMP pathways which, in addition to the MAPK pathway, are critical regulators of melanocyte proliferation [68]. PAK5 wildtype, S364L, and D421N expressing cells displayed similar levels of intracellular cAMP and showed no change in total or phosphorylated β-CATENIN, CREB or MITF (data not shown). However, using an antibody raised against phosphorylated substrates of the AGC kinases, PKA, PKC, and PKG, we found that hMELT cells expressing PAK5 S364L or D421N exhibited heightened activation of these pathways in comparison to wildtype control cells. Furthermore, this increase was eliminated in hMELT cells expressing PAK5 S364L-KD or D421N-KD. These results indicate that mitogenic PAK5 mutants hyperactivate AGC protein kinases to stimulate melanocyte proliferation (Figure 6A).

Based on these observations, we examined the effect of the PKA inhibitor, H89, on the proliferation of our hMELT stable cell lines. H89 is known to have off-target effects against other AGC kinases, but exhibits >10-fold selectivity for PKA over PKG and >500-fold over PKC [69]. Initial studies indicated that 10 µM H89 reduced AGC signaling by more than 50% in hMELT cells expressing wildtype PAK5 cells (Supplementary Figure 3A, 3B). *In vitro* kinase assays confirmed that this reduction in activity was not attributed to inhibition of PAK5 (Supplementary Figure 3C). Therefore, we performed proliferation assays, treating PAK5 wildtype, M173I, S364L, and D421N expressing cells with 10 µM H89 30 minutes prior to, and throughout, the eight-hour EdU labeling period. The proliferation rates of hMELT cells expressing PAK5 S364L or D421N were reduced following H89 treatment (Figure 6B, Supplementary Figure 3D, Supplementary Figure 3E). However, the H89-treated PAK5 S364L and D421N expressing cells still proliferated faster than wildtype control cells (*p* = 0.01). These results reveal the ability of PAK5 to regulate PKA signaling and demonstrate that the mitogenic activity of PAK5 S364L and D421N functions in part through increased PKA signaling.

## DISCUSSION

These studies establish PAK5 as the most frequently altered PAK-family member in human melanoma (Figure 1A). Based upon mutational rates published by TCGA, the 2,160 base pair PAK5 coding sequence should be randomly mutated in 3.6% of melanoma cases [1]. However, we find that 15% of melanomas contain a PAK5 missense mutation (Figure 1A), suggesting that alterations in this gene are advantageous to tumor development and/or progression. To elucidate these functions, we characterized the most frequently observed PAK5 mutants in human melanoma (E144K, M173I, E294K, S364L, and D421N). While *PAK5* alterations are rare in tumor types other than melanoma (Supplementary Figure 1A), the mutants characterized by our study appear in additional tumor sequencing datasets. Specifically, PAK5 E144K has been observed in colorectal adenocarcinomas, PAK5 S364L in lung adenocarcinomas, colon adenocarcinomas, and uterine endometrioid tumors, and PAK5 D421N in multiple lung adenocarcinomas [51, 70-73]. Beyond single nucleotide variants, 5.1% of melanomas exhibit elevated *PAK5* mRNA levels similar to the increased expression of other *PAK* family members in human cancer (Figure 1A) [33]. This observation implies that elevated PAK5 activity is advantageous to the tumor and prompted our investigation of potential gain-of-function phenotypes elicited by common, melanoma-associated *PAK5* missense mutations.

Our findings show that although the kinase activity of common, melanoma-associated PAK5 mutants is unaltered *in vitro*, a subset of these variants (PAK5 S364L and D421N) are able to promote melanocyte proliferation (Figures 2C, 4A, 5B, 6B). These data support a model in which PAK5 S364L and D421N engage mitogenic signaling pathways more effectively than the wildtype protein (Figure 6C). Such engagement could come in the form of increased affinity for wildtype substrates and/or the acquisition of novel binding partners. In accordance with this model, it remains possible that melanoma-associated PAK5 mutants alter single-cell migration and/or temozolomide resistance in the context of specific cancer proteomes. We see that a subset of PAK5 mutants are capable of stimulating ERK activation even in the absence of PAK5 kinase activity. However, the impact of this inappropriate ERK activation appears dependent upon how sensitive the cells are to MAPK-stimulated proliferation.

The PAK5 S364L and D421N mutants lie within a disordered and largely uncharacterized protein domain flanked by two proline-rich PxxP repeats (Figure 2A, orange boxes) [54, 74]. This domain extends from amino acids 351 to 445, is serine-rich (28%), and harbors 23% of all PAK5 missense mutations reported in the provisional TCGA melanoma dataset [51]. Our model predicts that PAK5 S364L and D421N alter the PAK5 interactome to promote melanocyte proliferation and is consistent with the conclusion that this serine-rich domain may be important for PAK5 protein interactions. This hypothesis is supported by a recent study showing that a region of PAK4, homologous to PAK5 amino acids 426-452, interacts with the Rho-family guanine nucleotide exchange factor, GEF-H1 [75]. Other work predicts that the PAK4 Q358 and R359 residues, which are conserved amongst group II PAKs and equivalent to PAK5 residues Q486 and R487, mediate substrate-selective differences between the group I and II PAKs [54]. Changing these amino acids to the corresponding PAK2 residues results in a PAK4 protein that preferentially binds group I, rather than group II peptide substrates [54]. These data suggest that the region surrounding these amino acids is critical for group II PAK substrate interactions. Furthermore, the PAK5 serine-rich domain shares 75% homology with other group II PAKs and mutations localized to this region are observed across multiple tumor datasets deposited in cBioPortal [51]. As PAKs interact with substrates involved in diverse tumorigenic processes, mutations that impact these protein:protein interactions may drive tumor progression. Notably, post-translational modifications in the serine-rich domain have yet to be documented and although the central region of PAK5 (aa109-420) is reported to elevate kinase activity through oligomerization, the catalytic activity of PAK5 S364L and D421N is unchanged (Figure 2C) [76, 77]. Taken together, these data support a model in which the unstructured, serine-rich domain proximal to the PAK5 kinase domain is important for mediating PAK5:substrate interactions and that alterations within this region can give rise to tumorigenic consequences.

Our findings show that PAK5 S364L and D421N alter intracellular signaling through both kinase-independent and -dependent functions. As an example of these kinase-independent functions, we find that the expression of either PAK5 S364L-KD or D421N-KD enhances ERK phosphorylation (Figure 5A). A similar kinase-independent phenotype, attributed to the improved scaffolding of proteins within the MAPK pathway, is reported in colon cancer cell lines overexpressing a kinase dead version of PAK1 [32]. While prior publications show that PAKs, including PAK5, can directly phosphorylate members of the MAPK signaling cascade [59-64], our data indicate that the kinase-independent functions of PAK5 S364L and D421N are sufficient to stimulate ERK activation in melanocytes. Nevertheless, the mitogenic effect of these mutants in hMELTs is maximized when catalytic activity is intact, and we identified PKA as one kinase-dependent pathway required to drive aberrant melanocyte proliferation (Figure 6A, 6B). While PKA is known to directly phosphorylate PAK1 and 4 in other cell types [78-81], our studies are the first to show that PAK5 functions upstream of PKA signaling and may reflect a regulatory pathway specific to melanocytes.

Only one other report has examined the role of *PAK5* missense mutations in cancer prior to our study. This publication focused on several lung-cancer-associated variants located near the serine-rich, unstructured domain (i.e. S312C, V463L, T538N, and V593I) [82]. Like PAK5 S364L and D421N, the authors showed that these mutants are capable of activating MAPK signaling; however, the ability of these proteins to drive aberrant proliferation in the presence or absence of kinase-activity was not explored. Nevertheless, these data bolster our hypothesis that the serine-rich domain of PAK5 engages mitogenic signaling pathways, and that mutations within this domain could enhance these interactions to drive cell cycle entry.

Efforts to develop effective PAK inhibitors have been hindered by poor specificity and high toxicity [43]. Our data suggest that targeting specific PAK interactomes may improve therapeutic results. Along this line, two prior studies identify peptides that block the interaction of PAK1 with SH3-domain proteins [83]. Use of these selective peptides prevents cellular transformation and migration in cell culture models. Taken together, these data provide the impetus to comprehensively characterize the PAK5 interactome and identify regions of the protein essential for pro-tumorigenic functions.

## MATERIALS AND METHODS

### Cell culture

Primary human melanocytes immortalized with hTERT and CDK4^R24C^ (hMELTs) [65], were maintained in Ham’s F12 supplemented with 7% FBS, 1% penicillin-streptomycin, 2 mmol/L L-glutamine, 50 ng/ml TPA, 100 µM IBMX, 1 µM Na_3_VO_4_, and 50 µM dbcAMP. HEK293T (ATCC CRL-3216) cells were cultured in DMEM supplemented with 5% FBS, 1% penicillin-streptomycin, and 2 mmol/L ʟ-glutamine. To generate stable cell lines, hMELTs were transduced with lentivirus in the presence of 10 µg/ml polybrene (Sigma #H9268). Stably transduced lines were selected and maintained in 1 µg/mL puromycin beginning two days after viral transduction.

Cell line identity was validated by short tandem repeat analysis (Michigan State Genomics Core) and cultures were regularly tested for mycoplasma contamination using Mycoplasma Plus PCR Primers (Agilent).

### Plasmid vectors and lentiviral production

pDEST-PAK5 was kindly provided by Dr. J. Brognard (CRUK Manchester). Wildtype *PAK5* was cloned from the pDEST vector into the *BamHI* and *EcoRI* sites of pLenti-puro using Gibson Assembly Master Mix (NEB, #E2611). The pLenti-puro vector is a derivative of pTRIPZ (Open Biosystems) in which turboRFP and rtTA3 were removed and a multiple cloning sequence inserted between the *Bam*HI and *Not*I restriction sites. pLenti-puro PAK5 mutants were generated using PCR mutagenesis and Gibson Assembly Master Mix (NEB, #E2611). PAK5 lentiviruses were made in HEK293T cells using standard procedures

### Immunoblotting

hMELT-PAK5 stable lines were grown to 70% confluence and then placed for 18 hours in media containing 2% serum and no additional growth factors. Cells were lysed in RIPA (150mM NaCl, 1% IGEPAL-CA-630, 0.5% deoxycholate, 0.1% SDS, 50mM Tris pH 8.0) containing protease (Sigma, P8340) and phosphatase (Fisher, 78420) inhibitors, separated by SDS-PAGE and transferred to a PVDF membrane. Resulting membranes were blocked in 5% milk and incubated with primary antibody overnight at 4°C. All primary antibodies are listed in Supplemental Materials (Supplementary Table 1). Washed membranes were incubated for 45 minutes at room temperature in secondary antibody solution (LI-COR IRDye 680, 800; 1:15000 in 5% milk), imaged on an Odyssey^®^ CLx, and quantified using Image Studio Software. *p*-values were calculated using multiple t-tests and significance determined by FDR (Q = 5%).

### *In vitro* kinase assays

PAK5 kinase activity was measured using ADP-Glo following the Promega protocol. Briefly, FLAG-PAK5 was immunoprecipitated from hMELT stable cell lines using anti-FLAG conjugated magnetic beads (Sigma #M8823). After washing the beads three times with 150 mM RIPA for 5 minutes on a rotator, purified FLAG-PAK5 was incubated with 0.2 mg/ml AKT (PKB) substrate (CKRPRAASFAE; SignalChem #A05-58), 50 µM UltraPure ATP, and Kinase Reaction Buffer A (40 mM Tris pH 7.5, 20 mM MgCl_2_, 0.1 mg/ml BSA) for 15 minutes at room temperature. The kinase reaction was quenched with ADP Glo Reagent for 40 minutes and then Kinase Detection Reagent was added for 30 minutes at room temperature. Luciferase activity was measured using a Veritas™ Microplate Luminometer with 1 second of integration. Raw Luciferase measurements were normalized to the amount of FLAG-PAK5 immunoprecipitated, which was quantified from immunoblots using Image Studio Software. *p*-values were calculated using multiple t-tests and significance determined by FDR (Q = 5%).

### Migration assays

hMELT stable cell lines were analyzed for migratory defects using a microfluidics device (20 µm width x 20 µm height x 700 µm length) (See Supplemental Materials for device manufacturing details). Microfluidic devices were treated with 10 µg/ml fibronectin for one hour. 600,000 cells were then introduced into the center seeding port of the microfluidics device containing normal growth media. The next day, cells were placed in media containing 0.1% serum and melanocyte growth factors for 18 hours. Full serum media was added to the outside collection ports to create a gradient. Images were taken every 5 minutes for 12 hours on a BioTek Lionheart FX Automated Microscope. The average velocity (µm/min) and persistence of single cells traversing the channels were calculated using the MTrackJ plugin for ImageJ [83]. Persistence was calculated for each cell by dividing the cellular displacement by the total distance traveled. *p*-values were calculated using multiple t-tests and significance determined by FDR (Q = 5%).

### Cell viability assays

30,000 hMELT stable cell lines were seeded in black-walled 96-well plates (Thermo Scientific #165305) coated with poly-L-lysine (Sigma #P1274). The next day, cells were treated with either DMSO or temozolomide (Sigma #T2577). Treatment media was changed every two days for a total of 7 days. On the 7^th^ day, cells were incubated with 0.0036% resazurin (Sigma #R7017) for one hour at 37°C and fluorescence measured in a BioTek Synergy™ HT microplate reader using 540/59 excitation/emission filters. *p*-values were calculated using multiple t-tests and significance determined by FDR (Q = 5%).

### EdU incorporation and flow cytometry

hMELT stable cell lines were seeded at equal density, placed for 18 hours in medium containing 2% serum and no additional growth factors, and then incubated with 10 µM EdU for 8 hours. Cells were harvested and processed as described in the Click-iT EdU Flow Cytometry Assay Kit (Invitrogen #C10634). Briefly, cells were fixed in ice cold 4% paraformaldehyde, permeabilized in 1x Invitrogen saponin buffer, and covalently labeled with 642 fluorescent azide (Active Motif #15288). Cells were run either on a FlowSight flow cytometer (Amnis) and analyzed using IDEAS software or on a BD Biosciences LSRII flow cytometer and analyzed using FlowJo Software. *p*-values were calculated using multiple t-tests and significance determined by FDR (Q = 5%).

When investigating the PKA signaling pathways, the hMELT stable cell lines were seeded and processed as described above except for one change. Cells were treated with either DMSO or H89 (Selleckchem #S1582) 30 minutes prior to and throughout the EdU labeling period. *p*-values were calculated using a paired t-test.

## SUPPLEMENTARY MATERIALS FIGURES, TABLE AND VIDEO





## Abbreviations

PAK: p21-Activated Kinase
hMELT: Human Immortalized Primary Melanocytes
AID: Auto-inhibitory Domain
TCGA: The Cancer Genome Atlas
