# Supplementary material for: Melanoma-associated mutants within the serine-rich domain of PAK5 direct kinase activity to mitogenic pathways
Source: Oncotarget. 2018 May 22;9(39):25386–401. doi: 10.18632/oncotarget.25356 (PMC5986637; doi:10.18632/oncotarget.25356)
Supplement: Supplementary file 1 [file oncotarget-09-25386-s001.pdf]

Melanoma-associated mutants within the serine-rich domain of PAK5 direct kinase activity to mitogenic pathways

SUPPLEMENTARY MATERIALS AND METHODS

Manufacturing of microfluidic devices used for migration assays

hMELT stable cell lines were analyzed for migratory defects using a custom Bi-directional Migration Assay (BMA) and time-lapse microscopy (Fig 3A). The BMA has an array of parallel aligned microtracks which were fabricated out of polydimethylsiloxane (PDMS) using standard photo- and soft-lithography [1]. Briefly, silicon wafers were piranha cleaned and then spin coated with SU-8 2025 (MicroChem). The spin coated wafers were then soft baked on a hot plate at 65°C for 1 minute followed by 2 minute heating at 95°C. The silicon wafer was then exposed to high intensity UV though a transparency mask thereby transferring the final pattern onto the wafers. The exposed wafer was then hard-baked at 65°C for 1 minute followed by 95°C bake for 3 minutes. Wafers were then treated in an SU-8 developer leaving behind the negative pattern of the final design onto the wafer. Finally, the wafers were hard baked at 200°C for 2 minutes. All the silicon wafer processing till this point was done in class 100 cleanroom. The Silicon wafers were silanized (T2492-KG, UCT Specialties) to passivate the wafers from sticking to PDMS. For replica molding final PDMS devices, a 10:1 mixture of elastomer to cross-linking agent (Slygard 184® Silicone Elastomer Kit, Dow Corning) was mixed, desiccated and then poured onto the silicon masters. The silicone mixture was cured at 65°C in a convection oven for 2 hours. The cured PDMS with the final patterned devices were then cut out from wafers and irreversibly plasma bonded to PDMS coated cell culture grade 6 well plates. The plates were sterilized for 60 minutes under high intensity UV making them suitable for cell culture experiments.

A

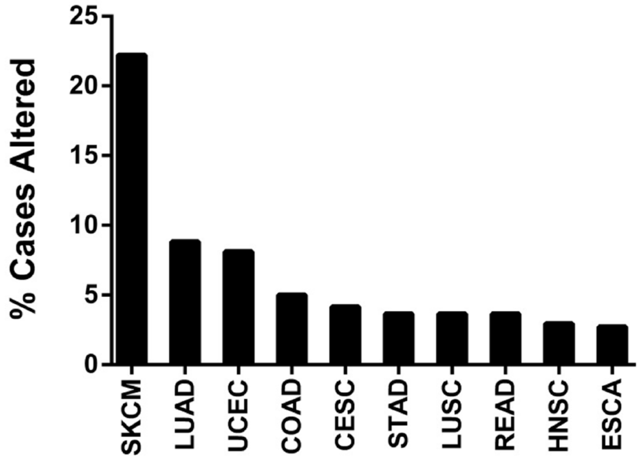

B

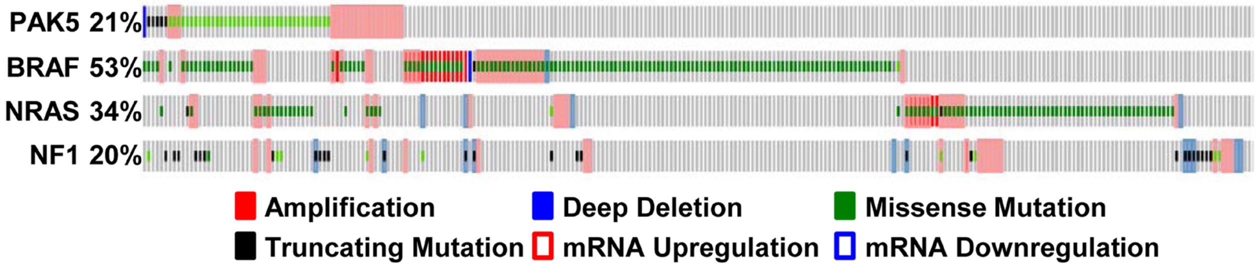

C

Gene #1	Gene #2	p-value	Log odds ratio	Relationship
**PAK5**	**NF1**	**0.005**	**0.913**	**Co-occurrence**
PAK5	BRAF	0.195	0.294	
PAK5	NRAS	0.303	0.198	
**BRAF**	**NRAS**	**<0.001**	**-2.226**	**Mutual exclusive**
**BRAF**	**NF1**	**<0.001**	**-1.027**	**Mutual exclusive**
NRAS	NF1	0.171	0.335	

D

Gene #1	Gene #2	p-value	Log odds ratio	Relationship
PAK5	PAK1	0.428	-0.238	
PAK5	PAK2	0.058	-1.046	
**PAK5**	**PAK3**	**0.024**	**1.042**	**Co-occurrence**
PAK5	PAK4	0.329	0.380	
PAK5	PAK6	0.402	0.232	

Supplementary Figure 1: PAK5 alterations are prevalent in melanoma and co-occur with alterations in NF1. A. The frequency of *PAK5* alterations in multiple TCGA tumor datasets analyzed using the NCI GDC Data Portal. SKCM = skin cutaneous melanoma, LUAD = lung adenocarcinoma, UCEC = uterine corpus endometrial carcinoma, COAD = colon adenocarcinoma, CESC = cervical squamous cell carcinoma and endocervical adenocarcinoma, STAD = stomach adenocarcinoma, LUSC= lung squamous cell carcinoma, READ = rectum adenocarcinoma, HNSC = head and neck squamous cell carcinoma, ESCA = esophageal carcinoma. **B.** cBioportal Oncoprint, generated from the TCGA dataset, depicting the co-occurrence of *PAK5* alterations with *BRAF*-, *NRAS*- and *NF1*-alterations in human melanoma. **C.** Results from Fisher's Exact tests assessing the co-occurrence and/or mutual exclusivity between *PAK5*, *BRAF*, *NRAS* and *NF1* alterations in melanoma. Bolded values are statistically significant. **D.** Results from Fisher's Exact tests assessing the co-occurrence and/or mutual exclusivity between *PAK* gene alterations in melanoma. Bolded values are statically significant.

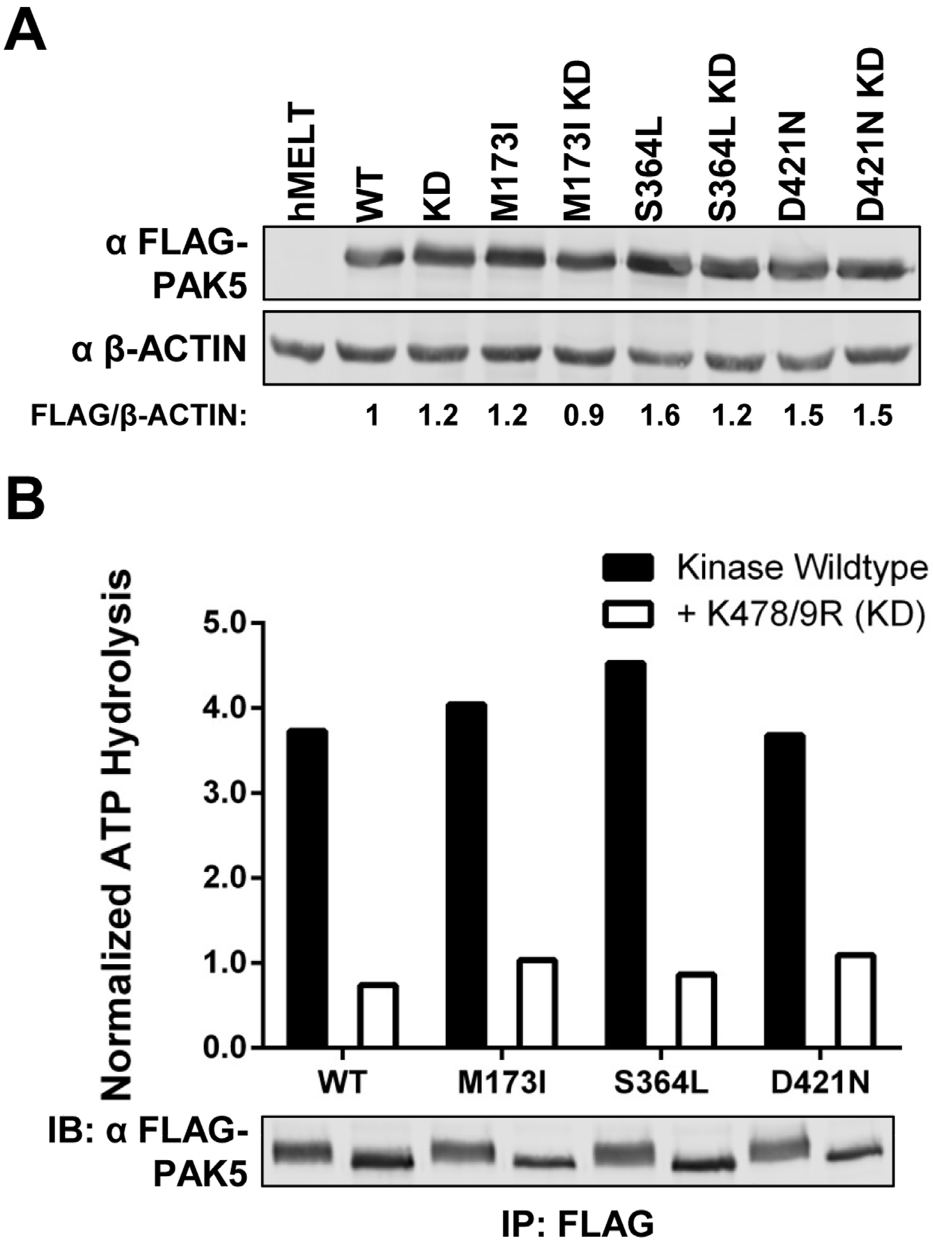

Supplementary Figure 2: Validation of kinase dead melanoma-associated PAK5 mutants. A. Immunoblots depicting the stable expression of FLAG-tagged wildtype and kinase dead (KD) PAK5 variants in hMELTs. 'hMELT' indicates the parental cell line. Shown below each lane is the β-ACTIN-normalized level of mutant PAK5 expression relative to wildtype (WT) PAK5. **B.** PAK5 kinase activity was measured using the ADP-Glo (Promega) system and normalized to immunoprecipitated protein levels as measured by immunoblot. Shown below is an immunoblot of immunoprecipitated FLAG-PAK5 samples used in the kinase assay.

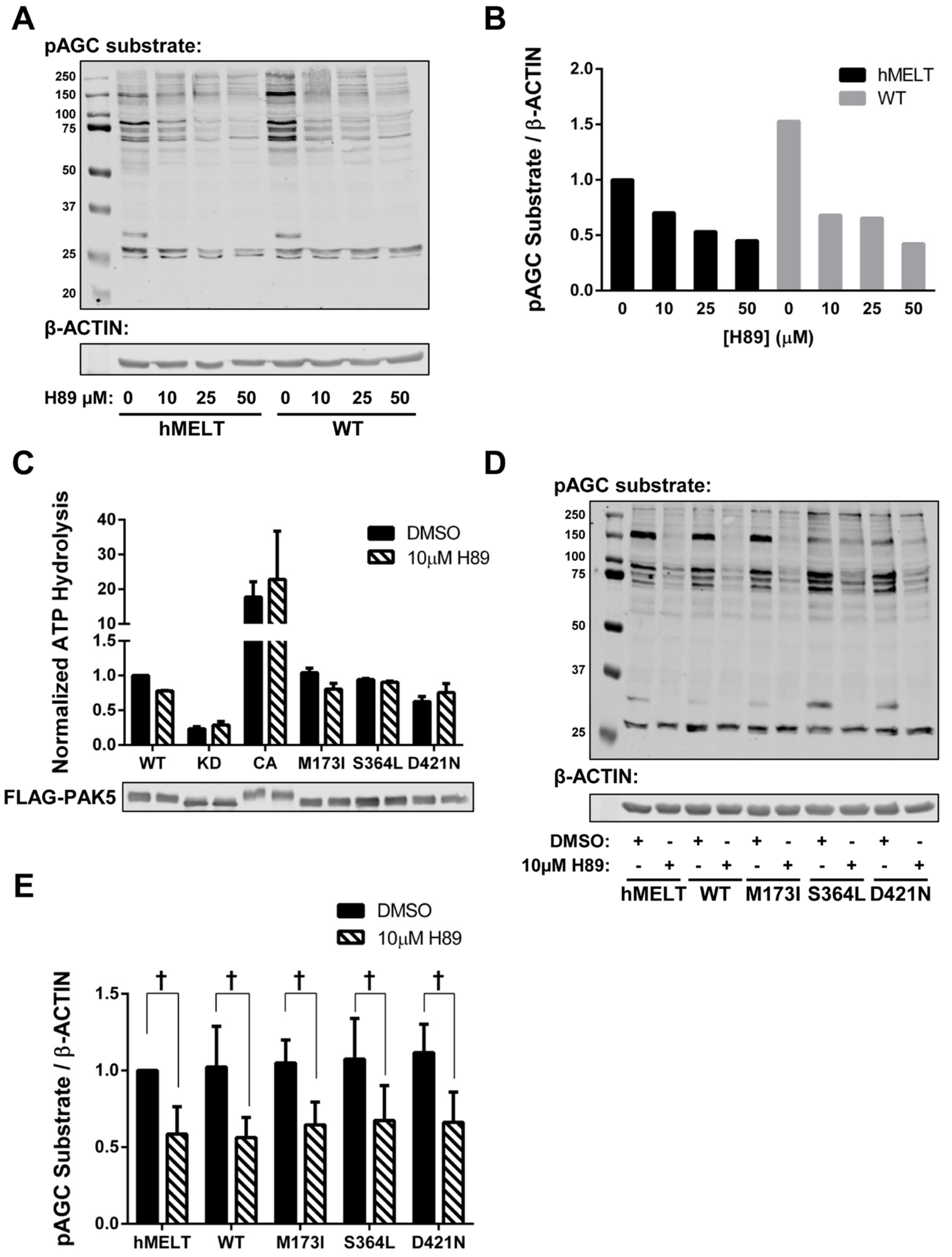

Supplementary Figure 3: H89 inhibits PKA signaling in hMELT stable cell lines. A. hMELT stable cell lines were placed in 2% serum for 18 hours and then treated with DMSO or increasing amounts of H89 for 8.5 hours. Lysates were then analyzed by SDS-PAGE followed by immunoblotting with an antibody that recognizes the phosphorylated motif of AGC kinases. 'hMELT' indicates the parental cell line. **B.** Quantification of each lane from the immunoblot represented in 'A' where values indicate the β-ACTIN-normalized, phosphorylated substrates of AGC kinases. **C.** hMELT stable cell lines were starved of serum and growth factors for 18 hours and then treated with DMSO or 10 μM H89 for 8.5 hours. PAK5 kinase activity was measured using the ADP-Glo (Promega) system and normalized to immunoprecipitated protein levels as measured by immunoblot. Shown below is a representative immunoblot of immunoprecipitated FLAG-PAK5 samples used in the kinase assay. Each bar represents the mean with error bars indicating the range. **D.** hMELT stable cell lines were incubated in 2% serum for 18 hours and then treated with DMSO or 10 μM H89 for 8.5 hours. Lysates were then analyzed by SDS-PAGE followed by immunoblotting for phosphorylated substrates of AGC kinases. 'hMELT' indicates the parental cell line. **E.** Quantification of each lane from the immunoblots represented in 'D' where values indicate the average signal of phosphorylated substrates of AGC kinases, normalized to β-ACTIN and relative to parental hMELT cells across at least three biological replicates. Error bars represent the standard deviation. †= $p < 0.05$ compared to each respective DMSO control. *p*-values were calculated using multiple t-tests and significance determined by FDR (Q=5%).

Supplementary Table 1: List of antibodies used in the study

Antibody	Company	Cat. #	Dilution
β-ACTIN	Cell Signaling Technologies	3700	1:5000
BRAF	Santa Cruz	sc-5284	1:1000
BRAF-pS445	Cell Signaling Technologies	2696	1:1000
CRAF	BD Biosciences	610151	1:1000
CRAF-pS338	Cell Signaling Technologies	9427	1:1000
ERK	Cell Signaling Technologies	4696	1:1000
ERK-pT202/Y204	Cell Signaling Technologies	4370	1:1000
FLAG	Sigma	F1804	1:1000
MEK1/2	Cell Signaling Technologies	9122	1:1000
MEK-pS217/221	Cell Signaling Technologies	9121	1:1000
PAK5	R&D Biosystems	MAB4696	1:1000
PKA-pSer/Thr Substrate	Cell Signaling Technologies	9621	1:1000
